# Substance Use Disorder–Related Hospitalizations and ED Use in Those Returning to Community From Prison in Later Life

**DOI:** 10.1093/geroni/igaa057.2691

**Published:** 2020-12-16

**Authors:** Lisa Barry, David Steffens, Kenneth Covinsky, Yeates Conwell, Amy Byers

**Affiliations:** 1 UConn Center on Aging, Farmington, Connecticut, United States; 2 University of Connecticut School of Medicine, Farmington, Connecticut, United States; 3 University of California San Francisco, San Francisco, California, United States; 4 University of Rochester, Rochester, New York, United States; 5 University of California, San Francisco / San Francisco VA Health Care System, San Francisco, California, United States

## Abstract

High rates of substance use disorders (SUDs) in persons age 50 and older are concerning. Those reentering the community in later life after incarceration are especially at risk. We determined if later-life prison release increases risk of SUD-related hospitalizations and ED visits in a national sample of veterans (N=7,671) released from prison between 2012 to 2014 and matched never-incarcerated controls (N=7,671). Later-life prison release was associated with increased risk of any SUD-related hospitalization/ED visit (2907.1 vs. 465.0 per 100,000/year; adjusted HR=2.67; 95% CI, 2.11-3.36) and 3-fold risk of hospitalizations/ED visits due to alcohol use disorder (1955.4.1 vs. 282.6 per 100,000/year; adjusted HR=3.04; 95% CI, 2.24-4.13) and drug use disorder (1586.1 vs. 252.0 per 100,000/year; adjusted HR=3.09; 95% CI, 2.23-4.30). Those reentering the community in later life after prison are at higher risk of experiencing SUD-related hospitalizations or ED visits. Prevention and intervention efforts targeting later-life prison-to-community care transitions are needed. Part of a symposium sponsored by the Aging, Alcohol and Addictions Interest Group.

